# Analysis of the profile of cardiovascular risk in Brazilian schoolchildren: metabolic and behavioral indicators

**DOI:** 10.20945/2359-3997000000269

**Published:** 2020-06-19

**Authors:** Daniela Casagrande, Alceu Afonso Jordão, Paulo Henrique Waib

**Affiliations:** 1 Centro de Pesquisa em Hipertensão e Metabolismo Faculdade de Medicina de Marília Marília SP Brasil Centro de Pesquisa em Hipertensão e Metabolismo, Faculdade de Medicina de Marília, Marília, SP, Brasil; 2 Departamento de Ciências da Saúde Faculdade de Medicina de Ribeirão Preto Universidade de São Paulo Ribeirão Preto SP Brasil Departamento de Ciências da Saúde, Faculdade de Medicina de Ribeirão Preto, Universidade de São Paulo, Ribeirão Preto, SP, Brasil

**Keywords:** Adolescent behavior, cardiovascular risk factors, cardiovascular diseases, prevention

## Abstract

**Objective:**

There is evidence demonstrating that cardiovascular diseases (CVD) manifesting during adulthood result from an intense interaction among risk factors that may have originated during childhood and adolescence. To compare the prevalence and clustering of cardiovascular risk factors in Brazilian schoolchildren with a 15-year interval between samples.

**Subjects and methods:**

A cross-sectional analysis based on the scores for cardiovascular risk factors was used to investigate 1,232 Brazilian schoolchildren of both sexes aged 12 to 18 years. The data of 596 schoolchildren of the 2000 sample were compared to those of 636 schoolchildren of the 2015 sample.

**Results:**

The prevalence of physical inactivity and abdominal obesity increased exponentially in both sexes from 2000 to 2015. The score for the clustering of cardiovascular risk factors showed that in 2000 the adolescents were exposed to 1 cardiovascular risk factor (31.7%), while in 2015 the greatest percentage was assigned to the category of 3 or more cardiovascular risk factors (34.9%), p < 0.001.

**Conclusion:**

The present results demonstrate a high prevalence of exposure to health risk behaviors of the adolescents studied over time. Considering the presence of modifiable risk factors, preventive measures regarding life style are essential.

## INTRODUCTION

Cardiovascular diseases (CVD) currently represent the first cause of death and premature disability in the world. In Brazil, CVD cause 30% of all deaths, 50% of which involving adults aged 30 to 60 years, i.e., in the full productive phase of life. This causes high public health costs due to hospitalization, procedures and early retirement ( 1 ).

The changes in the nutritional profile of the entire population evidenced in the last decades, known as the process of nutritional transition, are characterized by the decrease of innutrition and the increase of the rates of overweight and obesity in all ages, associated with the risk factors that predispose to the onset of CVD and social changes, economic and demographic effects of the country’s development process. The World Health Organization (WHO) proposes conducts for the control and prevention of risk factors linked to CVD in all age ranges, including childhood and adolescence ( 2 ).

The prevalence of overweight among adolescents has reached significant levels in recent years. At the beginning of the 1990s, 7.7% of Brazilian adolescents were overweight. In the 2000s, other studies developed in the country showed higher values. Data from the Family Budgets Survey (POF in the Portuguese acronym), carried out in 2002-2003, highlighted that 16.7% of adolescents aged 10 to 19 years were overweight ( 3 ). The National Survey on Demography and Health (PNDS) conducted in 2006 showed that 21.6% of adolescents aged 15 to 19 years were overweight and 4.4% of them obese ( 4 ). Data from one of the largest National Studies of Cardiovascular Risk in Adolescents (ERICA in the Portuguese acronym) ( 5 ) have pointed out that a significant parcel of young Brazilians aged 11 to 17 years show changes in plasma lipids, that 33.9% are overweight, and that 9.6% are already hypertensive.

The adolescence phase is considered to be a critical period for the beginning or persistence of habits that will reflect on the adult phase. Despite the importance of this topic, few studies have evaluated the clustering of cardiovascular risk factors (CVRF) in significant samples of young Brazilians. In view of the above considerations, the objective of the present study was to determine the prevalence and clustering of CVRF in representative samples of adolescent schoolchildren in the municipality of Marília, São Paulo, Brazil, over a period of 15 years in order to investigate the evolution of these factors over time.

## SUBJECTS AND METHODS

We analyzed cross-sectionally the anthropometric, pressure, glycemic and sociodemographic data collected in 2000 and 2015 of a representative sample of adolescent schoolchildren enrolled in public and private schools of Marília, a city located in the State of São Paulo, in the southeastern region of Brazil. It has a human development index of 0.798 and an estimated population of 230 thousand inhabitants ( 9 ,976 adolescents enrolled in high school in 2015). All reasonable efforts were made to identify a sample of schoolchildren that was representative of the population as a whole. The study was authorized by the Municipal Secretary Office of Education of Marília and was approved by the Research Ethics Committee of the Faculty of Medicine of Marília (CAAE 32063814.6.0000.5413), with financial support from the Coordination of Improvement of Higher Level Personnel (Capes in the Portuguese acronym).

Schoolchildren aged 12 to 18 years of both sexes whose parents or tutors gave written informed consent were included in the study. Adolescents with organic insufficiency, debilitating chronic diseases, type 1 diabetes mellitus or a physical/mental deficiency that would prevent their participation and pregnant girls were excluded from the study. The total number of participants was similar for the year 2000 (n = 596) and 2015 (n = 636).

Anthropometric measurements were obtained by the same group of trained evaluators in both years. The weight was measured with calibrated digital scale with capacity for 150 kg with precision of 0,1 kg, with the subject fasting, standing up with arms along the body, barefoot, and wearing light clothing. The height was determined by means of a fixed ruler on the wall with a scale in centimeters (2 meters in length with an accuracy of 0.1 cm) with students barefoot, standing straight on a smooth surface, with their back to the ruler and with feet close together. Body mass index (BMI) was calculated by the formula BMI = weight (kg)/height^2^ (m). Waist circumference (WC) was measured at the end of an expiration in the midpoint between the costal margin and the iliac crest using an inextensible anthropometric tape with 0.1 cm precision ( 6 ).

Nutritional status was defined on the basis of the BMI according to WHO criteria for adolescents: low weight (< 3rd percentile), overweight (≥ 85th < 97th percentile) and obesity (≥ 97th percentile), adjusted for sex and age ( 7 ).

Abdominal adiposity was defined according to the criteria of the International Diabetes Federation (IDF) for adolescents aged 12 to 16 years (≥ 90th percentile), adjusted for age and sex. The IDF criteria for adults were used for adolescents aged > 16 years (≥ 80 cm for women and 90 cm for men) ( 8 ).

Blood pressure was measured by the oscillometric method using an automatic device with accuracy from 0 to 299 mmHg and a cuff of the appropriate size for the arm circumference of adolescents. A single measurement was made with the subject sitting and with the left arm resting at the height of the heart after a 5-minute rest. Values ≥ the 90th percentile were considered for the diagnosis of high blood pressure in adolescents aged ≤ 16 years according to sex and height ( 9 ) and values ≥ 130 x 85 mmHg were considered for adolescents older than 16 years ( 8 ). Mean arterial pressure [MAP = SAP + (DAP x 2) / 3] was used for statistical analysis regarding its correlation with other risk factors.

Glycemia was determined in samples venous blood and capillary blood collected in the school environment itself after an 8 hour fast. For the comparison of the 2000 glycemia values (venous blood) and the 2015 values (capillary blood) we used a 15% correction of the capillary measure value ( 10 ). Hyperglycemia was diagnosed using a value ≥ 100 mg/dL or based on a confirmed diagnosis of type 2 diabetes mellitus ( 8 ).

The sociodemographic data were collected in a personal interview in the form of a standardized questionnaire, previously tested with questions regarding: socioeconomic data, regular smoking habit and alcohol consumption. Alcohol consumption was identified among the adolescents and was considered to be a risk behavior when the subject reported consumption during the last 30 days (as “yes” for 1 or more days). The level of physical activity was determined using the International Physical Activity Questionnaire (IPAQ) ( 11 ), short version. According to the IPAQ results, the subjects were divided into two major groups: active and physical inactivity.

A clustering score was used to define the presence of any combination of cardiovascular risk factors. For the analysis of continuous variables, the procedures of descriptive statistics were used and, subsequently, to identify any differences between genders, Student’s t-test. The score ranged from zero to 3 (zero = no exposure, 1 = exposure to one factor; 2 = exposure to two factors, and 3 = exposure to three or more risk factors). Descriptive statistical analysis of the data was processed with the R Core Team software, 2016. The chi-square test (χ^2^) was used to determine significant differences between categorical variables. Relative risk was determined in order to assess exposure and outcome, with a 95% level of significance of the confidence interval. The level of significance was set at 5% (p < 0.05) in all analyses.

## RESULTS

Of the 1,232 participating adolescents, 596 were studied in 2000 and 636 in 2015. The distribution of proportion was 54.2% *vs.* 60.7% for girls and 45.8% *vs.* 39.3% for boys. Mean age was 15.1 (± 1.1) *vs.* 15.3 (± 1.1) years). The baseline characteristics of the adolescents are described in table 1 .

**Table 1:** Mean values and standard deviation for information associated with risk factors predisposing to cardiovascular disease (n = 1,232)

	Total			Girls			Boys		
		
	2000	2015		2000	2015		2000	2015	
	**N = 596**	**N = 636**	p value*	**N = 323**	**N = 386**	p value*	**N = 273**	**N = 250**	p value*
BMI, kg/m^2^	21.6 (4.2)	21.5 (4.2)	0.822	21.7 (4.7)	21.8 (4.5)	0.827	21.4 (3.6)	21.0 (3.6)	0.299
WC, cm	73.5 (10.0)	77.0 (10.0)	< 0.001	72.2 (10.2)	76.2 (10.3)	< 0.001	74.9 (9.6)	78.2 (9.6)	0.001
SBP, mmHg	114.4 (12.3)	116 (17.2)	0.814	112.3 (11.7)	110.4 (10.5)	0.021	116.5 (12.9)	121.5 (13.5)	<0.001
DBP, mmHg	73.6 (8.5)	69.1 (9.5)	0.003	72.9 (8.2)	68.5 (8.8)	< 0.001	74.3 (8.8)	69.7 (10.3)	<0.001
Blood Glucose, mg/dL	93.7 (18.8)	89.0 (17.3)	0.103	92.9 (28.8)	87.5 (17.5)	0.170	94.6 (8.9)	90.4 (17.2)	0.052

* p value of the Student´s t-test.

BMI: body mass index; WC: waist circumference; SBP: systolic blood pressure; DBP: diastolic blood pressure.

The prevalence of CVRF is presented in table 2 . Comparison of the 2000 and 2015 samples revealed that the most prevalent factor was physical inactivity (66.5%) and the least prevalent was smoking (2.7%). There was an evident increase over time in the prevalence of abdominal adiposity physical inactivity and risky alcohol consumption.

**Table 2:** Proportion (n/%) of adolescents with factors predisposing to cardiovascular risk (2000 and 2015 comparison)

	Total			Girls			Boys		
		
	2000	2015		2000	2015		2000	2015	
**Risk factor**	**N = 596**	**N = 636**	p value*	**N = 323**	**N = 386**	p value*	**N = 273**	**N = 250**	p value*
Abdominal adiposity	179 (30)	305 (48)	< 0.001	131 (40.6)	221 (57.3)	< 0.001	48 (17.6)	84 (33.6)	< 0.001
Overweight	61 (10.2)	94 (14.8)	0.020	36 (11.1)	59 (15.3)	0.133	25 (9.2)	35 (14.0)	0.111
Obesity	48 (8.1)	55 (8.6)	0.784	26 (8.0)	40 (10.4)	0.354	22 (8.1)	15 (6.0)	0.455
Increased blood pressure	167 (28)	166 (26.1)	0.488	76 (23.5)	67 (17.4)	0.052	92 (33.3)	99 (39.6)	0.162
Hyperglycemia	72 (18,2)	135 (21.2)	0.278	36 (11.1)	68 (17.6)	0.347	36 (13.2)	67 (26.8)	0.726
Sedentarism	196 (32.9)	423 (66.5)	< 0.001	137 (42.2)	293 (75.9)	< 0.001	59 (21.6)	130 (52.0)	< 0.001
Smoking	81 (13.6)	17 (2.7)	< 0.001	35 (10.8)	7 (1.8)	< 0.001	46 (16.8)	10 (4.0)	< 0.001
Alcohol consumption	41 (6.9)	154 (24.2)	< 0.001	13 (4.0)	84 (21.8)	< 0.001	28 (10.3)	70 (28.0)	< 0.001

* p value of the chi-square test.

Altered blood pressure = values ≥ the 90th percentile according to sex and height. A value ≥ 130x85 mmHg was considered to be arterial hypertension for adolescents older than 16 years. Sedentarism: < 90 min/week physical activity^11^. Smoking = equivalent to smoking one or more cigarettes a day. Alcohol consumption = equivalent to the intake of one dose or more per day.

The proportion of adolescents with a cardiovascular risk according to sex in shown in table 2 . Physical inactivity was the most prevalent factor among girls in both studies (42.2% *vs* . 75.9%). In 2000, alcohol consumption (4.0%) was the risk of lowest percentage, while in 2015 the lowest risk factor was smoking (1.8%). Among boys, despite the lack of statistical significance, the most evident CVRF was the increase in blood pressure in 2000 (33.3%) and physical inactivity in 2015 (52.2%). In the 2000 sample, obesity was the lowest CVRF, while smoking was the lowest risk factor in 2015, as also observed for girls. When the data were stratified by sex, the increase in abdominal adiposity, physical inactivity and alcohol consumption continued to be the most important and significantly more prevalent CVRF.

The CVRF clustering score is presented in table 3 . No CVRF was observed in 17.3% of the entire sample investigated. When the score was stratified according to year of study, an impressive decline was noted in this proportion, with a significant decrease from 27.3% in 2000 to 7.9% in 2015. In 2000, the participants with 1 risk factors corresponded to the greatest percentage of the score (31.7%), whereas in 2015, the greatest percentage was of 3 or more risk factors ;4.9%). The analysis stratified by year and sex revealed a significant modification (p < 0.0001) of the prevalence of CVRF in both sexes. Among the 2000 girls, the highest percentages corresponded to categories 1 and 2 of the CVRF score (58.2%), whereas in 2015, 69.2% already belonged to the categories of 2 and 3 or more CVRF. Among the boys, the result was even more worrying, with 62.6% being classified into categories 0 and 1 of CVRF in 2000, as opposed to a majority of subjects (61.6%) with 2 or more CVRF in 2015 ( Table 3 ).

**Table 3:** Clustering scores for cardiovascular risk factors

Number of risk factors^a^	Group							p value*

	General n = 1,232		2000 Group n = 596		2015 Group n = 636			

	n	%	n	%	n		%	
0	213	17.3	163		27.3	50	7.9	0.001
1	354	28.7	189		31.7	165	25.9	
2	323	26.2	124		20.8	199	31.3	
3	342	27.8	120		20.1	222	34.9	

* p value of the chi-square test.

^a^ Clustering score for cardiovascular risk factors defined as the sum of any combination of the following factors: high blood pressure, hyperglycemia, smoking, excessive alcohol consumption, sedentarism, overweight and/or obesity and central obesity.

**Table 4:** Clustering scores for cardiovascular risk factors stratified by sex and year

Number of risk factors^a^	Girls					p value*	Boys				p value*
	
	2000 Grupo (n = 332)			2015 Group (n = 386)			2000 Group (n = 284)		2015 Group (n = 250)		
	
	n		%	n	%		n	%	n	%	
0	69	21.4		21	5.4	0.001	94	34.4	29	11.6	0.001
1	112	34.7		98	25.4		77	28.2	67	26.8	
2	76	23.5		128	33.2		48	17.6	71	28.4	
3	66	20.4		139	36		54	19.8	83	33.2	

* p value of the chi-square test.

^a^ Clustering score for cardiovascular risk factors defined as the sum of any combination of the following factors: high blood pressure, hyperglycemia, smoking, excessive alcohol consumption, sedentarism, overweight and/or obesity and central obesity.

**Figure 1:**
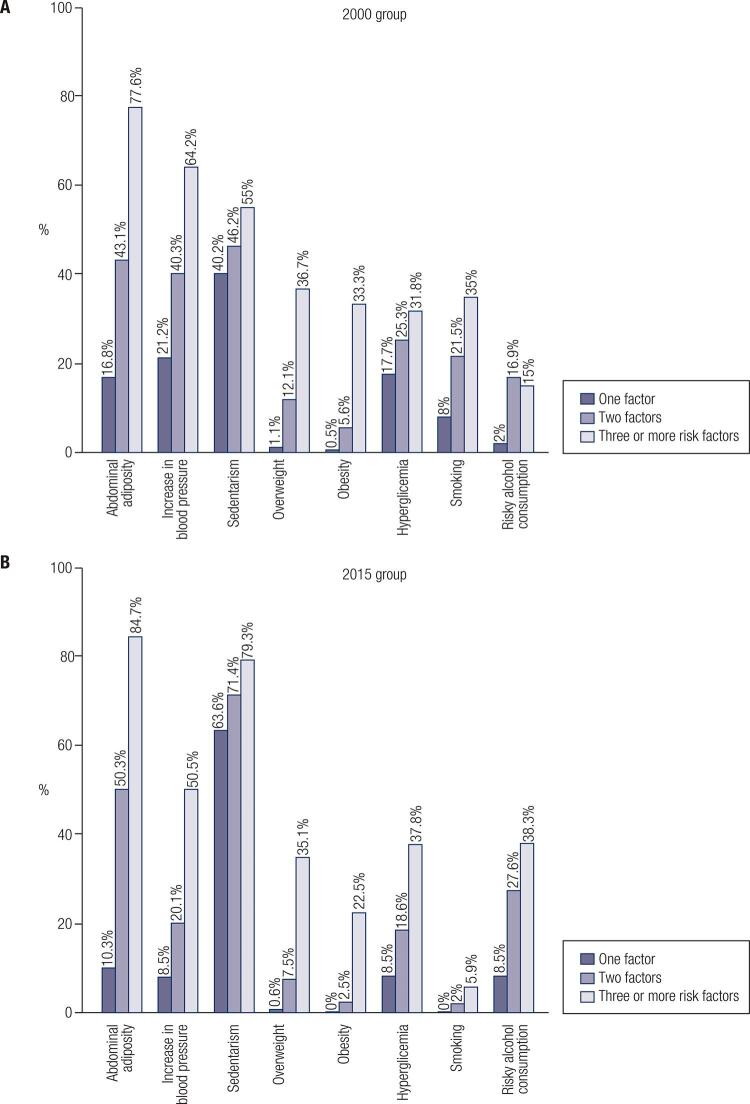
(A) Prevalence of each cardiovascular risk factors according to category of clustering score in 2000. (B) Prevalence of each cardiovascular risk factors according to category of clustering score in 2015.

Figure 1 illustrates the frequency of each risk factor within the score. Physical inactivity was the most prevalent factor in the categories of 1, 2 and 3 or more CVRF in both years. Abdominal adiposity was the most outstanding risk factor in both years, with a growing proportion. Among subjects with 3 or more risk factors, abdominal adiposity, increased blood pressure and physical inactivity were the most prevalent.

Analysis of the prevalence ratio did not reveal an association between physical inactivity and increased blood pressure. With similar results, both in 2000 and 2015, the adolescents with increased WC had a 1.8-fold higher risk (95% CI 1.39-2.39) to have an increase in blood pressure compared to those with WC within the indicated values.

## DISCUSSION

The present study describes the evolution of CVRF in samples of Brazilian adolescents over a period of 15 years. The risk factors associated with behavioral aspects such as physical inactivity and increased abdominal adiposity were the most prevalent. As a source of concern, the data show a modification of the lifestyle of adolescents with possible deleterious consequences associated with obesity such as dyslipidemia, cancer, arterial hypertension, atherosclerotic diseases, and nonalcoholic fatty liver disease ( 12 ).

The CVRF score revealed a high prevalence of health risk behaviors among adolescents over the years, as also reported in other similar studies all the world ( 13 ). The results of this survey indicate that in 2000 most adolescents were exposed to 1 cardiovascular risk factor, whereas in 2015 the highest percentage of adolescents reached the category of 3 or more CVRF. Sanchez and cols. ( 14 ), reported that almost 80% of US adolescents aged 11 to 15 years presented health risk behavior and only 2% met guidelines for physical activity and healthy eating. Hardy and cols. ( 15 ) reported that more than half of those assessed had 3 or more cardiovascular risk factors. With similar results, Plotnikoff and cols. ( 16 ), analyzing Canadian adolescents aged 11-17 years, found that 43% of boys and 53% of girls had 2 or more cardiovascular risk factors. The greater the amount of clustered risk factors, the higher the probability of the early onset and development of cardiovascular diseases ( 13 ).

Another result to be highlighted is the modification of the number of adolescents with no CVRF from 2000 (27.3%) to 2015 (7.9%). Other authors have also observed a low percentage of adolescents with CVRF ( 13 ) suggesting that sedentary adolescents tend to adopt simultaneously an inadequate diet and the use of alcohol and tobacco. In addition to deaths due to cardiovascular accidents, more than one third of cancer deaths in the world are attributed to modifiable risk factors such as smoking, physical inactivity, alcohol and tobacco consumption, inappropriate eating habits, and excess weight ( 17 ), these being the most prevalent factors in the p resent study. Among the modifiable causes of cancer in Brazil, 3.8% of all annual diagnoses are attributed to excess weight, with an increase to 4.6% predicted by 2025 ( 18 ). Although this information involves another age range, it becomes relevant due to the difficulty to change habits among children and adolescents.

Several studies ( 19 - 20 ) have reported a 10% to 25.9% variation in the prevalence of excess weight among adolescents. A significant change in excess weight (excess weight + obesity) from 18.3% in 2000 to 23.4% in 2015 (p < 0.27) was observed in the present study. In the United States, estimates from the National Health and Nutrition Survey (NHANES IV) ( 21 ) showed that in the early 2000s, in the 12-19 age group, overweight increased from 14.8% to 18.3% in boys and from 14.8% to 16.4% in girls. At the same time in Brazil, the prevalence of overweight in the 10 to 19 years’ age group was 18.0% in boys and 15.4% in girls ( 3 ). Adiposity rates also increased from 30% to 48% in 2015. These results agree with other Brazilian studies which showed that the prevalence rates of abdominal adiposity have been increasing over the last decades in this age range, varying from 27.3% to 47.6% ( 22 ). When compared to international findings, these values are higher than those of Spanish (11.6%) ( 23 ) and Polish (34,5%) ( 24 ). The accumulation of abdominal fat is known to be more dangerous by being more functionally active than the accumulation of subcutaneous fat and by having higher rates of lipolysis and being associated with insulin resistance ( 25 ).

The accumulation of adipose tissue detected by WC measurement proved to have an early influence on the blood pressure of the students. Previously published studies on adolescents have also identified associations between abdominal obesity and high blood pressure, although, when considered separately, blood pressure did not change over time. Comparison of the two measures has pointed out that WC measurement is an important tool for the assessment of visceral obesity since it is easy to obtain and is an important predictor of cardiovascular risk in adolescents, appearing to better estimate intra-abdominal fat than the BMI ( 26 ).

On average, 25% of the adolescents evaluated here in the two samples showed pre- or full arterial hypertension. In a meta-analysis study, Gonçalves and cols. ( 27 ) reported an 8.0% prevalence of systemic arterial hypertension among adolescents, and the ERICA ( 5 ) study reported a value of 9.6%, with a 28.4% prevalence among obese subjects. Several studies conducted in various Brazilian cities have reported different prevalence values of arterial hypertension among young persons. In Belo Horizonte, Ribeiro and cols. ( 20 ) detected a prevalence of 12%, in Rio de Janeiro ( 28 ) the prevalence was 19.4% and in Londrina it was 9% ( 13 ). We believe that the measurement of blood pressure on a single occasion may explain these different values. Although several studies perform only one measurement in order to diagnose arterial hypertension, the recommendation is to perform more than one since measurements obtained on a single occasion may not reflect the real values of adolescents, suggesting that multiple measurements on different occasion may have a reducing effect on arterial hypertension ( 29 ). In the present study, the same technique and interpretation of the results were used for both samples, supporting the reliability of the results.

In this study, we did not find an increase in blood glucose when comparing the samples. In a longitudinal study, Reinehr and cols. ( 29 ) reported that hyperglycemia can be the main abnormality among obese children and adolescents, thus contributing to dyslipidemia. The chronic inflammation caused by adipose tissue plus insulin resistance over time may trigger micro- and macrovascular complications due to vascular remodeling with the proliferation of cells of the vascular musculature, i.e., endothelial dysfunction, macrophage migration, increased platelet aggregation with consequent thrombus formation, and the risk to develop diabetes mellitus ( 30 ).

In Brazil, the prevalence of adolescents with insufficient levels of physical activity ranges from 39% to 93,5% ( 31 ). In the present study, the prevalence of physical inactivity was 32.9% in 2000, as opposed to double this value (66.5%) in 2015. With a similar study design and instrument for assessment, two student samples were evaluated in the city of Pelotas in 2005 and 2012, with a respective prevalence of physical inactivity of 69.6 and 69.9% ( 32 ). We believe that the 15-year interval between our studies was decisive for the notable changes in the practice of physical activity by the adolescents. In study, Beck and cols. ( 19 ) detected a prevalence of physical inactivity was 61,2%. Among the possible causes of the increased rates of physical inactivity is the process of urbanization. The arrival of the technology era and the facilities due to modernization may have influenced the change in life style of the population, especially regarding the practice of physical activity.

In the present study, in contrast to a decrease in tobacco consumption, alcohol consumption increased exponentially among the adolescents studied from 6.9% to 24.2%. Data obtained in the large Brazilian studies, PeNSE (National Survey of School Health) ( 33 ), conducted on Brazilian schoolchildren have shown that 26.1% of the subjects interviewed, presented risk behaviors regarding the regular consumption of alcoholic beverages. In addition to having a social impact, alcohol consumption in this age range is a source of concern because of damage to neurocognitive development and the potentiation of the risk of cardiovascular diseases.

Studies with objectives similar to those of the present one have warned that if the obesity trends are maintained in the long term, the prevalence for 2015 will be 11.3 million, with 427 thousand having pre-diabetes, 150 thousand type 2 diabetes, one million arterial hypertension, and 1.4 million stage 1 nonalcoholic hepatic steatosis ( 34 ). In an attempt to control the obesity epidemic, several countries, including Brazil, have adopted policies for the control of weight gain among children and adolescents. The Brazilian Association for the Study of Obesity and Metabolic Syndrome (Abeso) ( 35 ) recommends weight monitoring for children and adolescents based on BMI and WC in addition to the encouragement of the practice of physical activity and of the reduction of calorie intake.

In summary, we conclude that cardiovascular risk factors can originate in adolescence and, despite the implementation of various actions against obesity, still represent a challenge regarding the transformation of evidence into effective practices and the establishment of directives, especially in low income countries like Brazil. As a limitation of the present investigation is the different ways of assessing blood glucose in both periods. Finally, we emphasize further studies are needed aiming at the early prevention of cardiovascular diseases in this age range.
